# In-flow real-time detection of spectrally encoded microgels for miRNA absolute quantification

**DOI:** 10.1063/1.4967489

**Published:** 2016-12-06

**Authors:** David Dannhauser, Filippo Causa, Edmondo Battista, Angela M. Cusano, Domenico Rossi, Paolo A. Netti

**Affiliations:** 1 Center for Advanced Biomaterials for Healthcare@CRIB, Istituto Italiano di Tecnologia (IIT), Largo Barsanti e Matteucci 53, 80125 Naples, Italy; 2 Interdisciplinary Research Centre on Biomaterials (CRIB) and Dipartimento di Ingegneria Chimica, dei Materiali e della Produzione Industriale, Università degli Studi di Napoli “Federico II,” Piazzale Tecchio 80, 80125 Naples, Italy

## Abstract

We present an in-flow ultrasensitive fluorescence detection of microRNAs (miRNAs) using spectrally encoded microgels. We researched and employed a viscoelastic fluid to achieve an optimal alignment of microgels in a straight measurement channel and applied a simple and inexpensive microfluidic layout, allowing continuous fluorescence signal acquisitions with several emission wavelengths. In particular, we chose microgels endowed with fluorescent emitting molecules designed for multiplex spectral analysis of specific miRNA types. We analysed in a *quasi*-real-time manner *circa* 80 microgel particles a minute at sample volumes down to a few microliters, achieving a miRNA detection limit of 202 fM in microfluidic flow conditions. Such performance opens up new routes for biosensing applications of particles within microfluidic devices.

## I. INTRODUCTION

MicroRNAs (miRNAs) are a class of short (approximately 18–23 nucleotides), single-stranded, non-coding endogenous RNAs that play important roles in regulating gene expression via target miRNA degradation or translational repression (Bartel, 2004). It has been demonstrated that the expression levels of circulating miRNAs are correlated with the different states of various diseases including cancer, neurodegenerative disorders, and cardiovascular diseases (Schwarzenbach *et al.*, 2014; Grasso *et al.*, 2014; and Sayed *et al.*, 2014). However, an accurate and robust quantification of circulating miRNA biomarkers in blood for early stage, metastatic or recurrent diseases is still a major challenge due to high sequence homology, complex secondary structures, and low concentration levels.

Furthermore, due to the low amount of target miRNAs, large volumes of clinical samples—from a hundred to several millilitres—are typically required, which only few conventional detection systems can directly handle without sample preparation and volume reduction. Moreover, analytical sensitivity and specificity are further hindered by a generally low signal to noise ratio (SNR). In particular, even though the quantitative reverse transcription polymerase chain reaction (qRT-PCR) method shows high sensitivity (down to 10 fM) and high-throughput ability, it requires miRNA extraction, amplification, and calibration steps (Chen *et al.*, 2011). More recently, a number of new methods including droplet digital PCR (ddPCR), nanopore-based detection, sequencing, and electrochemical sensing methods have also been developed for the detection of miRNAs (Hindson *et al.,* 2013; Campuzano *et al.*, 2014; Duan *et al.*, 2013; Wang *et al.*, 2011; Labib *et al.*, 2013; Das *et al.*, 2015; and Murtaza *et al.*, 2013). However, all of them require pre-treatments as well as amplification steps of the sample.

Novel suspension arrays offer the advantages of higher flexibility by simply changing/adding probe particles, enhanced reaction kinetics due to radial diffusion, shorter incubation and assay times, lower sample consumption, and lower costs (Evans *et al.*, 2003; Nolan and Sklar, 2002; Chapin *et al.*, 2009; Al-Ameen and Ghosh, 2013; Wang *et al.*, 2005; and He *et al.*, 2007). Suspension arrays involving polystyrene beads doped with combinations of dyes for optical coding have been developed by Luminex (Birtwell and Morgan, 2009). Moreover, suspension array technologies with barcodes, Au/Ag nano-barcodes, silica nanotubes, dot-coded polymer particles, and Ilumina's VeraCode have been developed to further improve their multiplexed readouts (Chapin *et al.,* 2009; Al-Ameen and Ghosh, 2013; Wang *et al.*, 2005; He *et al.,* 2007; and Birtwell and Morgan, 2009). Recently, particle-based suspension arrays have been attracting increasing interest for the multiplexed detection of nucleic acids, offering high flexibility, easy probe-set modification, and high degrees of reproducibility (Wilson *et al.*, 2006). Among particles, microgels feature a flexible molecular architecture, antifouling properties, and enhanced sensitivity with a large dynamic range of detection (Aliberti *et al.*, 2016). Such platforms improve diffusion kinetics while keeping the benefits of hydrogel suspension assays, decrease assay times while reducing non-specific adsorption and retaining hydration thus improving the capturing of oligonucleotides, thanks to the bio-inertness of water-laden hydrogels (Zhao *et al.,* 2015).

Our research group developed microgels with different fluorescent dyes—for multiplex spectral analyses—and endowed with fluorescent probes for the specific detection of miRNAs (Causa *et al.,* 2015). In particular, a double strand displacement assay was mounted onto the microgel network, where a fluorescent tail had preliminarily quenched while a fluorescence recovery occurred after target capture. Moreover, the sub-micrometric size of the microgels contributed to the enhancement of fluorescence detection due to the confinement in a small volume. The antifouling properties of the microgels also permitted the direct measurement of miRNAs in serum. Even though the approach proved more accurate than qRT-PCR, the need to manipulate optical focus and record fluorescence images from hundreds of microgels is difficult and time consuming (Lim *et al.*, 2014). Instead, a trend towards non-Newtonian fluids, which allow viscoelastic 3D particle migration towards a centreline in straight or circular microchannels, can be noticed (Lim *et al.,* 2014; Romeo *et al.,* 2013; D'Avino *et al.*, 2012; Del Giudice *et al.*, 2015; and Kang *et al.*, 2013). In fact, viscoelastically induced particle alignment—at low volume concentrations—permits the optical observation of target particles in a simple and cost-effective way (Dannhauser *et al.*, 2014 and Dannhauser *et al.,* 2015).

Furthermore, the current techniques allow converting the recognition event from single particles into electrical or optical signals for detection, but these techniques require sophisticated equipment and trained staff for detection and analysis (Lin *et al.*, 2016). The platforms with electrical signals are easy to use and inexpensive, but the detection limit is poor, whereas the platforms with optical signals offer improved sensitivity but require sophisticated analysing protocols, which restrict the applications for real-time online detection. Until now, the development of simple and ultra-sensitive detection platforms for real-time online detection of miRNA by microgels has remained a challenge.

Here, we present a straightforward implementation of a *quasi* real-time fluorescence detection of single microgels in microfluidic flows using non-Newtonian fluids at a low concentration level. In particular, a pressure-driven viscoelastic solution was used to manipulate microgels with non-overlapping fluorescence emissions related to both spectral barcodes for multiplex analysis and absolute quantification of miRNAs. To prove the concept of a multiplex assay, different barcodes corresponding to different emissions at specific wavelengths (505–530 and 550–600 nm) were used. Instead, the fluorescent recovery, at another wavelength (650–750 nm), occurred after double strand displacement upon specific target capture. The miRNA concentration had been previously calibrated to convert fluorescence intensity at 670 nm in a concentration with ultra-sensitive resolution. We investigated miRNA 21 (miR21) as a proof of principle since it has been related to the pathogenesis of various malignant tumours, including prostate, gastric, colon, breast, and lung cancers (Di Girolamo *et al.*, 2016).

## II. MATERIAL AND METHODS

### A. Microgel synthesis

Fluorescent encoded microgels were prepared through a multistep procedure, combining a free-radical precipitation polymerization and a general seeding polymerization with different amounts of fluorescent molecules in the feeding solutions as well as adapting the synthesis to the in-flow detection (Causa *et al.*, 2015 and Battista *et al.*, 2016).

The barcodes were provided with a fluorescent core, a separation shell, and an alternative fluorescent shell on top. The poly-ethylene glycol (PEG) cores were prepared by co-polymerization of 1% (w/vol.) total monomer concentration of PEG-dimethacrylate (PEGDMA, Mn 550, SIGMA-ALDRICH) and different amounts of methacryloxy thiocarbonyl Rhodamine B (Rh), namely, 0.3, 0.2, and 0.1 mM. Afterwards, two subsequent shells were added: first, the Rh labelled cores were used as seeds for polymerization of 0.5% PEGDMA (first shell); then, a second fluorescent shell was added on top by copolymerizing PEGDMA, Acrylic Acid (AAc), and a different amount of Fluorescein O-methacrylate (Fluo), namely, 0.2 mM, 0.1 mM, and 0.05 mM. Such combination allows obtaining nine different barcodes as reported in Table S1 of the supplementary material.

**TABLE I.** Fluorescence ratios and concentrations of the investigated microgel mix. The relative amount of each code for quiescent as well as in-flow measurements is shown with the corresponding acquisition channel (B-CH). Non-related microgels were excluded from the results.

Code	Fluo (mM)	Rh (mM)	Initial	Quiescent^a^	In-flow^a^	B-CH
1	0.20	0.30	30%	26.3 ± 2.4%	27.1 ± 1.6%	2
2	0.10	0.10	10%	4.3 ± 1.1%	6.5 ± 0.9%	5
3	0.05	0.10	60%	60.8 ± 3.0%	46.2 ± 2.7%	6, 7

^a^ Average of three measurements.

The reactions were initiated by potassium persulfate—at temperatures above 60 °C—and the obtained microgels were washed several times by centrifugation and dialysis. The probes were then integrated into the microgel molecular network. In particular, based on the miR21 sequence (23 nucleotides), we designed the displacement assay probes formed by (i) a tail (12 nt), labelled with Cy5 at the 5′ end, modified with an amine group on the 3′ position for covalent immobilization on the microgel and (ii) a quencher strand (23 nt) internally modified with a Black Hole Quencher (BHQ), partially complementary to the tail and fully complementary to the miR21 target. When the quencher strand and tail partially hybridize, Cy5 fluorescence quenching occurs. In the presence of the target, the quencher and the miR21 target hybridize, so that the Cy5 fluorescence emission is recovered (as shown in detail in Fig. S1 of the supplementary material). The length of the tail was optimized to obtain an appropriate difference in free energy (20 kcal mol^−1^) between the tail-quencher and the target-quencher duplex (see supplementary material, Table S2).

**FIG. 1.**
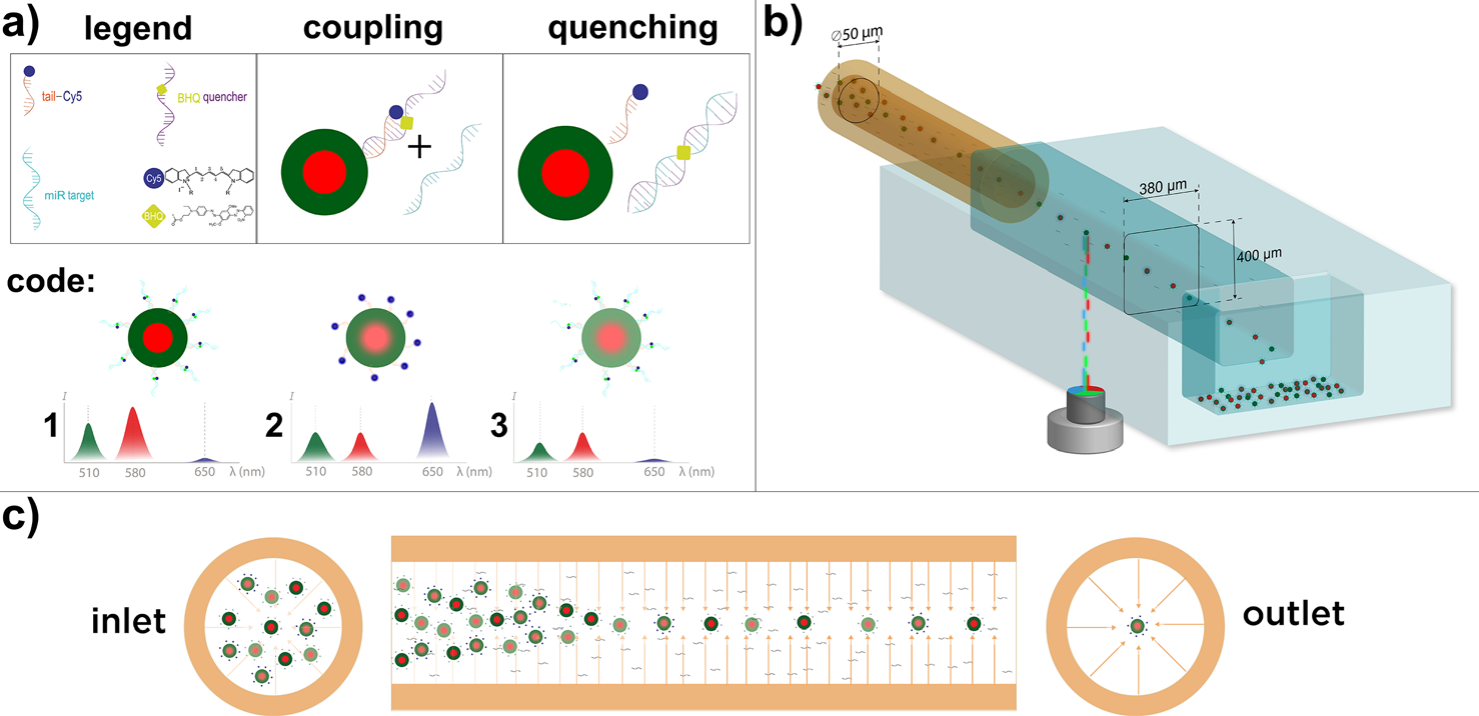
Microgel barcode with the microfluidic alignment and readout system. (a) The coupling and quenching process of the barcode system is illustrated and three different codes are shown with typical corresponding fluorescence emission spectra below, where receptors of code 2 are bound with miR21 sequences. (b) The readout system, where a round glass capillary enters the measuring channel of 380 × 400 *μ*m from the side, providing the 3D alignment of particles along the central axis. Three alternating excitation wavelengths strike the passing particles from below, exciting the corresponding fluorescence barcodes. Measured particles are collected in a reservoir positioned at the end of the channel. The ferrule connecting the capillary to the channel and the PMTs for signal detection are not shown for readability. (c) The viscoelastic alignment principle where microgel migration to the centreline of the round capillary over distance is shown.

### B. Sample preparation

For the following steps, a saline buffer solution consisting of 10 mM Tris HCl pH8 (150 mM NaCl) was used. 50 *μ*g of microgels coupled with specific tail-Cy5 DNA (250 *μ*l buffer) were mixed with 75 pmol of specific quencher-DNA (250 *μ*l) and incubated overnight at room temperature. Afterwards, the microgels were washed and re-suspended in 100 *μ*l of saline buffer solution to obtain a final concentration of 0.5 *μ*g *μ*l^−1^.

Experiments were investigated by measuring different ratios of the barcoded microgels as well as absolute miRNA concentration levels to verify the accuracy of our detection approach. Doing so, each detected code corresponded to a specific ratio between the emission intensities of Fluo *vs*. Rh and the tail-Cy5 DNA. We investigated a microgel mix consisting of three different codes (1, 2, and 3) for our tests. In addition, for the in-flow quantification of the absolute miR21 concentration, a separate calibration curve was obtained using miR21 concentrations ranging from 10^−15^ to 10^−7^ M, while for the final experiments 1 pM of miR21 solution was added to the microgel mix.

Quiescent measurements were performed by loading 50 *μ*l of about 0.05 *μ*g *μ*l^−1^ of microgel suspension in a glass channel (μ-slide, IBIDI) and imaged after a settlement time of about 1 h. In the case of in-flow measurements, the suspension (0.5 *μ*g *μ*l^−1^) was 100 times diluted in a viscoelastic alignment solution consisting of 0.6 g of poly-ethylene oxide (PEO-M_w_ = 4 MDa, SIGMA-ALDRICH) dissolved in 1 dl of saline buffer solution. The microgel size was determined with a standard dynamic light scattering apparatus (3D Light Scattering Spectrometer, LS INSTRUMENTS).

### C. Microgel alignment

Viscoelastic induced particle alignment is of considerable interest, due to the fact that the spatially non-uniform viscoelastic properties of a non-Newtonian polymer solution under Poiseuille flow trigger particles to migrate in the centreline without any external force (Romeo *et al.*, 2013; D'Avino *et al.*, 2012; Kim and Kim, 2016; Yuan *et al.*, 2015; Lu *et al.*, 2015; and Nam *et al.*, 2015). In general, a non-Newtonian fluid shows two different rheological characteristics under deformation, one related to the viscous fluid component and the other to the elastic solid behaviour. Indeed, the alignment of a pressure-driven viscoelastic solution strongly depends on its elastic part. Lateral particle migration in polymer solutions in square channels has been studied due to its practical importance in lab-on-a-chip applications. We have chosen a cost effective polymer, which generally shows reduced relaxation times compared to DNA as the alignment trigger (Kim and Kim, 2016).

Here, we used a microfluidic system designed ad-hoc adapted for the most common microscope types. Such a system consists of four parts: a round glass capillary, a pressure pump, a microfluidic channel, and a soft ferrule, resulting in a simple and inexpensive particle alignment system. The inner capillary radius *r_c_* was set to a value of 25 *μ*m, while the channel was constructed with a cross section and length of 380 × 400 *μ*m and 40 mm, respectively. A minimum capillary length *L* of 500 mm was used for our system due to constructional restrictions of the used microscope. Furthermore, we employed a ferrule to seal the microfluidic channel entrance and to connect the capillary to the microfluidic channel. Finally, a reservoir at the end of the microfluidic channel collected all measured microgel particles (see Fig. 1(b)).

### D. Fluorescence signal readout

A confocal microscope (TCS STED CW, LEICA), used in a modality resonant scanner (8 kHz), was used for the readout of microgel codes and concentration levels of target miRNAs in microfluidic flows. In particular, three different excitation wavelengths (488, 543, and 633 nm) were alternated each 80 ms during the measurements (see Fig. 1(b)). Corresponding photo-multiplier tubes (PMTs) ranging from 505 to 530 and 550 to 600 nm were used to encode the fluorescence barcode spectra, while another PMT ranging from 650 to 750 nm was used to quantify the concentration level of miR21 on each microgel. A 25 × 0.95 water immersion objective was used to investigate the microgel particles passing in the microfluidic channel. A measurement window of 512 × 200 pixels—with a pixel size 0.202 × 0.202 *μ*m—was evaluated for each recorded PMT channel. We decided to use a confocal microscope, as it is commonly employed in biotechnology; however, a flow cytometry-based system or other and less sophisticated optical tools could be used (Calin and Croce, 2006).

The obtained data were analysed in *quasi* real-time by a self-written Matlab routine to encode the microgels as well as to achieve the corresponding concentration levels of target miRNAs. Thereby, for the in-flow microgel readout, each PMT signal obtained from a microgel passing a fixed pixel area of interest (pixel 50–80 in flow direction) was analysed separately and the intensity-ratios between Fluo and Rh (Fluo/Rh) and the corresponding normalized Cy5 intensity were calculated. The pixel area width of 30 pixels was chosen based upon the final flow conditions of the microgels. Indeed, for quiescent reference measurements, intensity-ratios were calculated over the whole measurement window of 512 × 512 pixels. Using the intensity-ratios between the individual PMT signals significantly reduced errors caused by slightly out-of-focus aligned microgels. The average intensity of 121 pixels centering the centroid-position of the obtained microgel in the pixel area of interest was calculated. The multicolour Fluo, Rh, and Cy5 signals of the investigated microgel mix are shown in Fig. S2 of the supplementary material. The mix of three different codes is illustrated in Fig. 1(a) with the corresponding fluorescence amount and wavelength below.

**FIG. 2.**
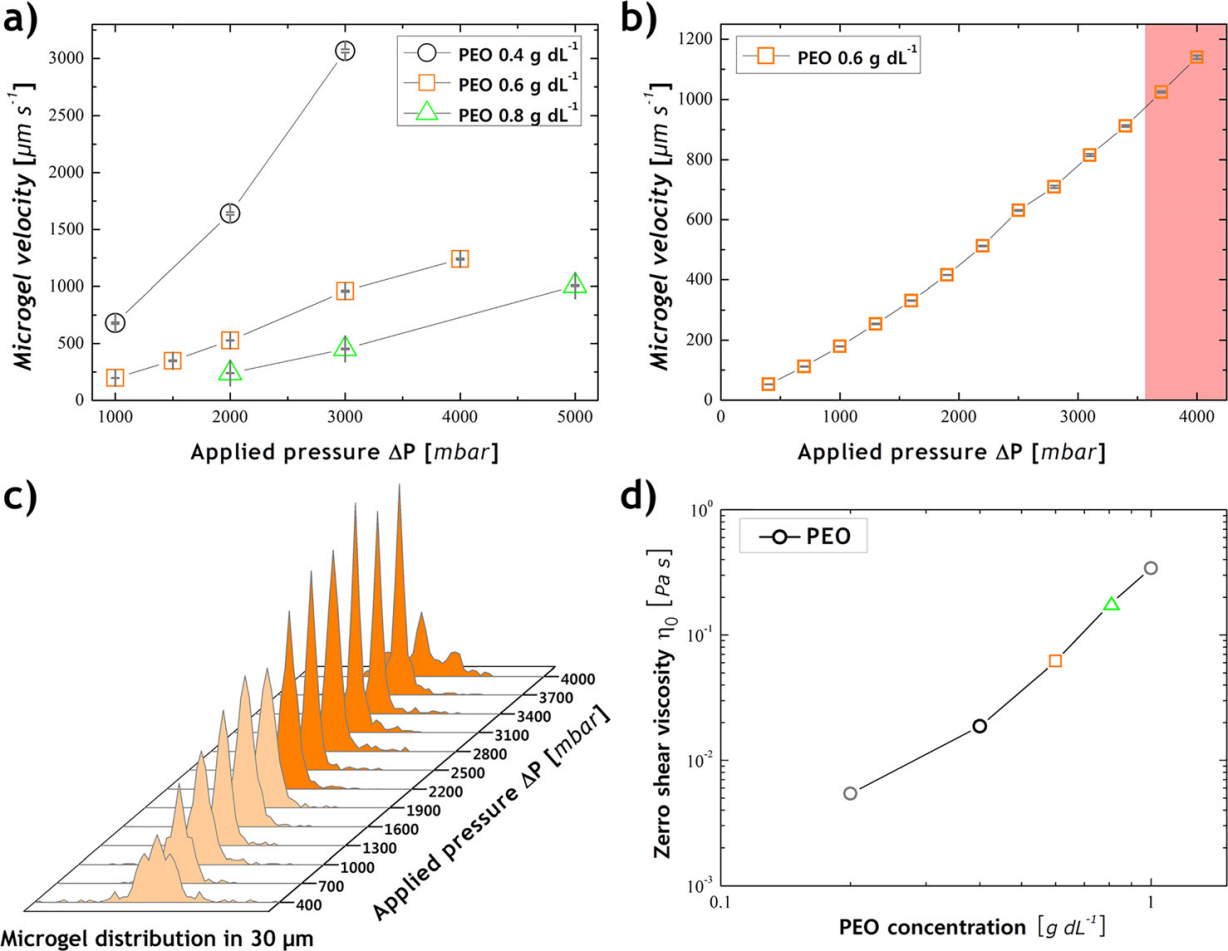
Microgel velocity and transversal distribution for varying levels of applied pressure. (a) Different viscoelastic polymer concentrations are investigated vs. microgel velocity and applied pressure. (b) The microgel velocity of PEO 0.6 g dl^−1^ was observed over a wide range of applied pressure levels, where the red area illustrates the zone with blurring effects in the signal readout. (c) The transversal alignment distribution of the microgel flowing in the microfluidic channel. The faded zone indicates the insufficient aligned microgel conditions. (d) The zero shear viscosities of various polymer concentrations are plotted.

## III. RESULTS AND DISCUSSION

### A. Microgel fabrication

We adapted spectrally encoded displacement assays with a core-shell structure. The cores were based on a co-polymer of PEG and Rh, which were used as seeds first for a PEG shell and then for a co-polymer of PEG-co-acrylic acid doped by different amounts of Fluo. The Fluo/Rh-ratio represents the spectral encoding. The miRNA target probes were integrated into the microgel molecular network, based on the miR21 sequence. A number of nine distinguishable barcodes were produced (see supplementary material, Table S1).

We designed the probes formed by (i) a tail labelled with Cy5 at the 5′ end, modified with an amine group on the 3′ position for covalent immobilization on the microgel and (ii) a quencher strand internally modified with a (BHQ), partially complementary to the tail and fully complementary to the target. When the quencher strand and tail partially hybridize Cy5 fluorescence quenching occurs. In the presence of the target, the quencher and the target hybridize so that the Cy5 and BHQ are away and the fluorescence emission is recovered (see Fig. 1(a)). The length of the tail was optimized to obtain an appropriate difference in free energy (20 kcal mol^−1^) between the tail-quencher and the target-quencher duplex (see supplementary material, Table S2). The bioconjugation was in the order of 10^4^ DNA probes/microgel. An average microgel radius *r_m_* of 0.61 *μ*m was achieved.

### B. Microfluidic condition

For the microgel barcode manipulation in a straight microfluidic channel, we investigated the use of viscoelastic PEO solutions. The probability of viscoelastically induced rigid particle alignment in the centreline of a capillary can be expressed by a dimensionless parameter $\theta =\dot{\gamma }\lambda_{t}+\beta^{2}(L/r_{c})$, with $\dot{\gamma }=(\Delta Pr_{c})/\left(8\eta_{0}L\right)$ the average shear rate of the alignment fluid, *ΔP* the applied pressure, *η*_0_ the zero shear viscosity of the viscoelastic medium, and *β* the confinement ratio between *r_m_* and *r_c_*. Consequentially, *β* was calculated to be 0.024, a smaller value compared to optimal alignment conditions reported in the literature (Romeo *et al.*, 2013 and Del Giudice *et al.*, 2015). However, a sufficient alignment condition (*θ* ≥ 1) can be simply achieved by an appropriate setting of the remaining previously mentioned parameters. Fig. 1(c) illustrates viscoelastically induced microgel alignment in the round capillary of the microfluidic system. Microgels are randomly distributed at the inlet of the capillary; when the distance increases, the microgels get directed to the centreline of the capillary before passing through the rectangular measurement channel.

We explored the best suitable microgel alignment conditions by varying the viscoelastic polymer concentration levels as well as ΔP values for the given microfluidic device parameters. Microgel velocities for alternating viscoelastic polymer concentration levels -spanning from 400 up to 4000 mbar of ΔP-measured by our readout system are presented in Fig. 2(a). Such results imply the usage of PEO 0.6 g dl^−1^ as the microgel alignment solution, due to the more suitable velocity range over a large *ΔP* range. Indeed, more diluted concentration levels result in significant higher microgel velocities with insufficient alignment results; on the other hand, higher concentration levels are limited by the performance of the used pressure pump and the required capillary length.

In addition, we investigated the most efficient microgel velocity range for the PEO 0.6 g dl^−1^ solution according to the maximum applicable velocity (see Fig. 2(b)) and best transversal alignment conditions (see Fig. 2(c)). We determined that useful microgel velocities up to 1000 *μ*m s^−1^ can be set before the blurring effects by the signal readout of the diverse PMT can be recognized. A sufficient transversal alignment could be obtained for *ΔP* over 2000 mbar. Aside from that no microgel deformation in the microfluidic channel could be recognized during all our measurements.

To calculate the microgel alignment probability, we measured *η*_0_ for various PEO concentrations using a standard Rheometer (MCR 302, ANTON PAAR, cone-plate geometry) and consequential calculated *θ* (see Fig. 2(d)). We found that for the indicated PEO and *ΔP* parameters the alignment conditions presented by Romeo *et al.* were fulfilled (Romeo *et al.*, 2013). All alignment calculations were based on stiff particle structures, while microgel structures can be assumed to be soft, which leads to an enhanced alignment probability (Yang *et al.*, 2012).

**FIG. 3.**
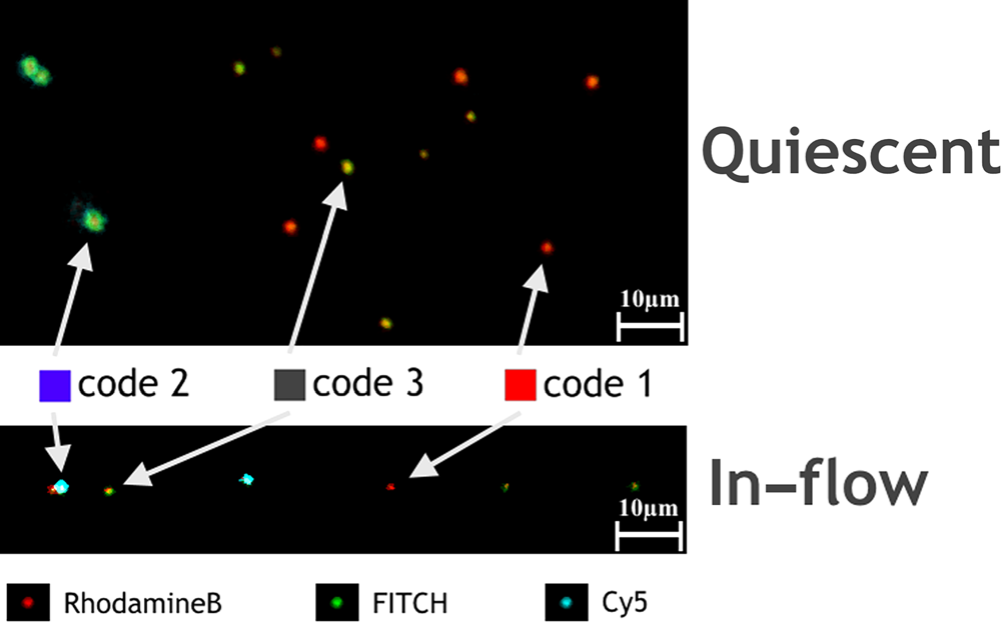
Multiplex microgel based miR21 target detection. Quiescent measurements are performed in a 512 × 512 pixel field of view, while 512 × 200 pixels are used in-flow. The flow direction is from left to right. The PMT signal for each separate fluorescence signal is overlaid according to the colour code shown at the bottom and its intensity.

We investigated a multiplex microgel solution encoded with different Fluo/Rh ratios, in which one microgel barcode possessed the capture oligonucleotide molecules for the specific detection of miR21. In fact, such variable fluorescence concentration levels were chosen to distinctively encode the microgels in microfluidic flow conditions. A typical comparison of a quiescent with an in-flow measurement is presented in Fig. 3, where each obtained fluorescence acquisition is represented according to its specific colour code. The combination of more than one colour code and its relative intensities results in the visually distinct barcodes. Beside the presented microgel codes, Fig. 3 also shows the viscoelastically induced microgel migration to the centreline of our microfluidic device.

### C. Quiescent measurement

First, we performed quiescent microgel investigations of the presented microgel mix using a glass μ-slide instead of the microfluidic device. Three separate acquisitions were performed adding all detected microgels to a final quiescent result, where the optical measurement parameters were set equal to in-flow measurements. In particular, 84 microgels were detected and analysed from measurement routine resulting in the reported barcode distributions presented in Table I.

### D. In-flow measurement

Afterwards, three separated in-flow acquisitions, each lasting 60 s using our microfluidic device, were performed and added to a unique in-flow result. In those courses, 230 microgels were measured and analysed with our measurement routine and compared to the quiescent results. During a measurement time of 3 min approximately 2.1 *μ*l of sample were analysed. It can be noted that in-flow acquisitions showed slightly lower absolute intensity values, which did not affect our measurement results, due to the fact that our routine calculated the relative fluorescence ratios to detect microgel types. Concerning this matter, a SNR above 20 dB was detected for all in-flow investigated microgel particles.

Detailed results from quiescent and in-flow measurements indicated that the majority of microgel codes was appropriately detected in their specific barcode channels, where increasing ratio values from left to right were plotted.

### E. Barcode channels

**FIG. 4.**
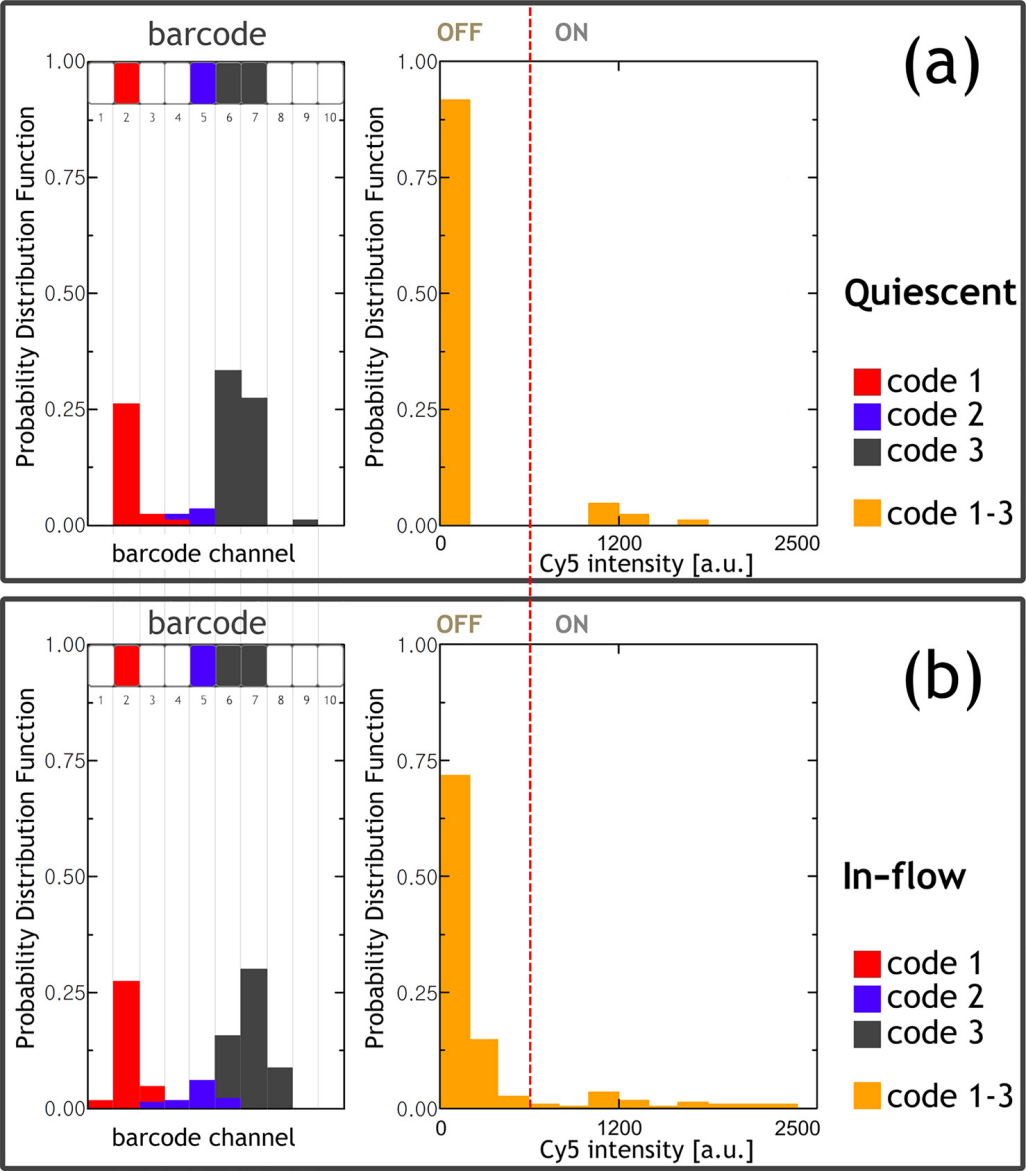
The quiescent and in-flow microgel readout results according to barcode channel and Cy5 intensity are presented. The abscissa of the barcode channel represents the Fluo/Rh ratio for each measured microgel, while the Cy5 intensity shows the normalized miRNA concentration level. The red dashed line indicates the threshold for the miR21 detection given by the LOD value. (a) Quiescent measurements of 84 microgel particles including all three codes are illustrated, while below in (b) 230 microgel particles measured in-flow are presented.

**FIG. 5.**
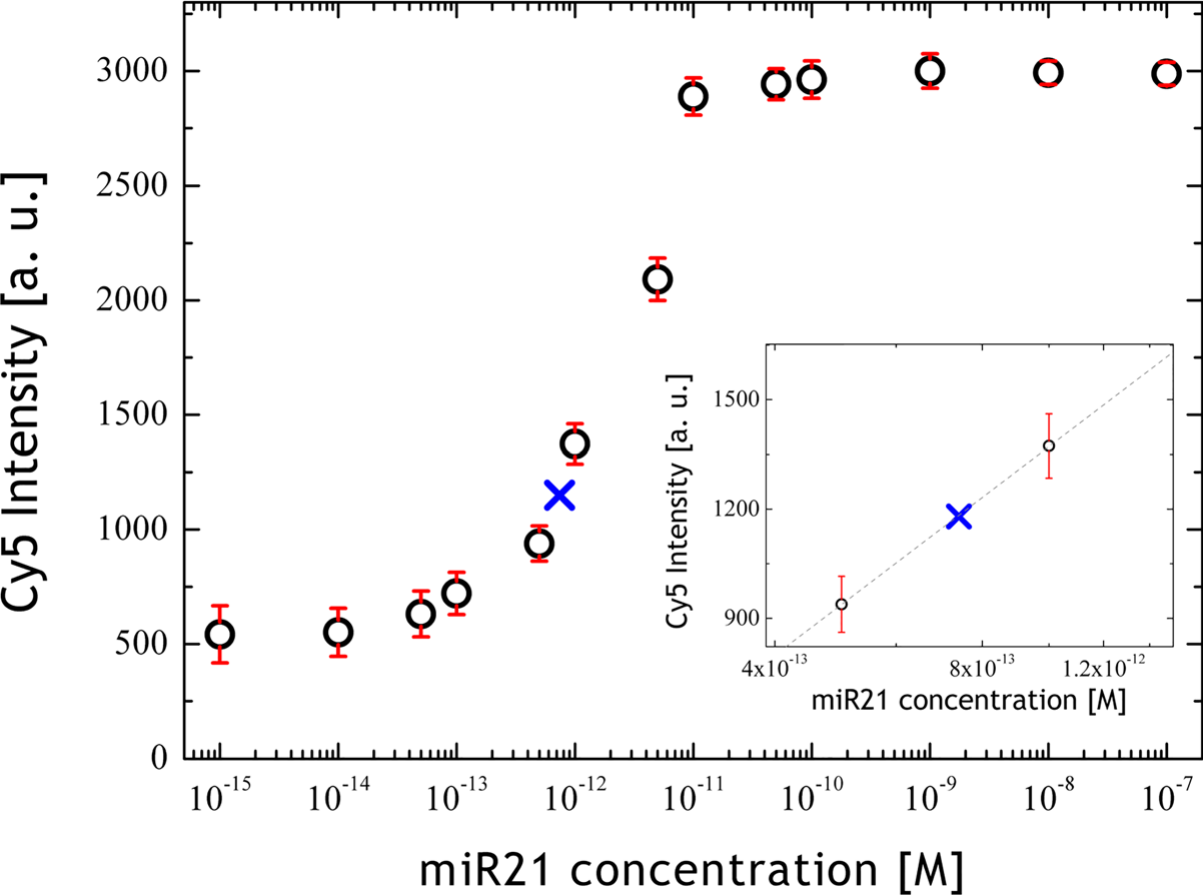
In-flow detection of miR21 over a dynamic range concentration level of 10^−15^–10^−7^ M used as look-up-table for obtained Cy5 intensities. The blue cross indicates the detected average Cy5 intensity of in-flow measurements, highlighted in more detail in the inset.

The barcode detection ratios were classified in similarly divided channels, where a higher Rh amount leads to smaller barcode channel numbers and *vice versa*. The abscissa of the miRNA detection indicates the Cy5 fluorescence intensity values normalized over Rh intensity and classified in similarly divided channels. Such a data normalization implies that each absolute measured Cy5 intensity above an intensity threshold -as illustrated in Fig. 4 with a red dashed line- given by the limit of detection (LOD) value for miR21 detected by a calibration measurement (see Fig. 5) is multiplied by a factor $x_{n}=\bar{Rh}/Rh_{n}$. Such a factor takes into account the mean Rh intensity ($\bar{Rh}$) of all obtained microgel particles and the absolute *Rh_n_* intensity values of the corresponding microgel particles, where *n* indicates the detected particle number. Such a simple procedure compensates for out-of-focus microgel particles and therefore miRNA concentration levels can be inferred for an optimal microfluidic flow position.

Microgels with code 3 display the highest Rh content compared to their Fluo amount, while their absolute Rh content is the lowest of all three used codes. Because Rh is generally better detected by PMTs, its content mainly determines the final barcode channel position. Microgels with code 1 have similar relative Fluo/Rh ratios compared to particles with code 3, but were detected at distinct lower barcode channels, due to their significantly higher absolute Rh amount. This also implies that microgels with code 2 having an intermediate Fluo and Rh content were detected in between barcode channels. In fact, such distinct barcode channel positions allowed us to generate a multiplex readout system in-flow, where we recognized no significant barcode overlapping between the measured microgel codes.

### F. Limit of detection

For the purpose of automatized miRNA detection in-flow, we obtained a calibration curve for miR21 and interpolated the absolute Cy5 intensity over the whole miRNA detection range; afterwards, we used these values as a look-up table for the detection of miRNA amounts (see Fig. 5). As a result, we calculated a LOD of about 202.1 fM for a ΔP 2000 mbar and a polymer concentration of 0.6 g dl^−1^. With such a look-up table, a final check for the absolute miR21 quantification was possible, resulting in a detected miR21 concentration of 0.74 pM, which is consistent with the initial concentration of 1 pM (indicated by a blue cross in Fig. 5). However, the LOD value can be further improved by increasing the conjugation yield of fluorescent tails (not beyond the point where electrostatic interactions and hindrance between oligonucleotides increase too much) or decreasing the microgel size (not under a volume lower than optical focus) (Causa *et al.*, 2015).

We also tested other miRNA types, resulting in similar outcomes (data not shown). Microgels exceeding the LOD threshold value of the normalized Cy5 intensity values were checked and resulted as belonging only to the microgel code 2, which is the only suitable microgel type for miR21 detection. Such an approach opens up the possibility to continuously record several target miRNA contents of independent sample volumes.

Our in-flow readout system is able to detect miRNAs in a multiplexed assay with a particle speed of up to 1000 *μ*m s^−1^, without significant loss of accuracy compared to the quiescent readout procedure. In such a way, we are able to analyse up to several differently encoded microgels in the course of 1 s, demonstrating the possibility to detect the relative amount of each utilized barcode.

## IV. CONCLUSIONS

We demonstrated the workability of a readout system using individual microgels in microfluidic flow conditions, able to continuously detect, count and identify specifically barcoded microgels, obtain fluorescence acquisitions, and calculate absolute quantification of specific miRNA concentration levels. Our approach is based on multiplex spectral analyses of individual predefined microgel barcodes endowed with an absolute readout of fluorescent probes for the detection of miRNAs, where no previous RNA sequence amplification is required, thus reducing any sources of measurement errors.

A cost-effective and biocompatible viscoelastic fluid was used to achieve optimal microgel alignment in the centreline of a straight channel. In such a way, a robust and simple readout system from up to nine microgel barcodes was assembled. Since the specific target detection of microgels can be easily tuned, the system can be applied to a wide range of different biomarkers thanks to its barcode structure. Moreover, the readout speed can be adjusted by the user according to the performance of any microscopic system, conferring high versatility to the proposed approach. We identified and counted hundreds of microgels in-flow conditions with different codes and quantified a specific miRNA target, demonstrating the specificity of the assay in multiplex measurement conditions. A miR21 concentration of 0.74 pM was detected in-flow, which is consistent with the initial sample concentration level.

## SUPPLEMENTARY MATERIAL

See supplementary material for the compositions of the synthesised barcodes, their specific DNA and RNA sequences and obtained confocal microscope investigations in-flow.
